# Reliable Inference of the Encoding of Task States by Individual Neurons Using Calcium Imaging

**DOI:** 10.1523/ENEURO.0378-25.2025

**Published:** 2026-01-26

**Authors:** Huixin Huang, Garima Shah, Hita Adwanikar, Shreesh P. Mysore

**Affiliations:** ^1^Department of Psychological and Brain Sciences, Johns Hopkins University, Baltimore, Maryland 21218; ^2^The Solomon H. Snyder Department of Neuroscience, Johns Hopkins School of Medicine, Baltimore, Maryland 21218; ^3^Kavli Neuroscience Discovery Institute, Johns Hopkins University, Baltimore, Maryland 21218

**Keywords:** calcium imaging, encoding, medial prefrontal cortex, neuron, selectivity, task states

## Abstract

Investigations into the neural basis of behavior frequently employ calcium imaging to measure neuronal activity. Across studies, however, seemingly reasonable but highly diverse methodological choices are typically made to assess the selectivity of individual neurons to task states. Here, we examine systematically the effect of parameter choices, along the pipeline from data acquisition through statistical testing, on the inferred encoding preferences of individual neurons. We use, as an experimental testbed, calcium imaging in the medial prefrontal cortex of freely behaving mice engaged in a classic exploration-avoidance task with animal-controlled state transitions, namely, navigation in the elevated zero maze. We report that most of the key parameters in the pipeline substantially impact the inferred selectivity of neurons and do so in distinct ways. Using novel accuracy and robustness metrics, we directly compare the quality of inference across combinations of parameter levels and discover an optimal combination. We validate its optimality using resampling methods and demonstrate its generality across the two common analytical approaches used to assess neuronal selectivity—average response rate-dependent selectivity indices and continuous time-dependent regression coefficients. Together, our results not only identify an optimal parameter setting for reliably assessing encoding preferences of cortical excitatory neurons using GCaMP6f calcium imaging but also establish a general data-driven procedure for identifying such optimal settings for other cell types, brain areas, and tasks.

## Significance Statement

This study addresses a critical unmet need in investigations of the neural basis of behavior with calcium imaging, namely, a standardized set of parameter values for the reliable assessment of neuronal selectivity for task states. By objectively evaluating the impact of various parameter choices on the inferred selectivity of excitatory neurons (in the mPFC of freely behaving mice using GCaMP6f), it identifies an optimal parameter combination that yields accurate and reliable inferences. This combination is calcium events convolved with a 2 s exponential decay filter, head-centric animal position data, 50 ms binning of data, animal-controlled dataset sizes for task states, and randperm shuffling for statistical testing.

## Introduction

Understanding the neural representations underlying distinct task states is a major goal of systems and behavioral neuroscience. A frequently asked question in this context is whether and to what extent individual neurons in a brain area preferentially encode one state over another. For instance, studies have investigated whether individual neurons differentially encode social interactions (Frost et al., 2021; Fustiñana et al., 2021; Nair et al., 2023), action-related behaviors (e.g., rear, forward, and left-turn; Klaus et al., 2017), occupancy of safe versus aversive spatial regions (Adhikari et al., 2011; Jimenez et al., 2018; Gründemann et al., 2019; Lee et al., 2019; Johnson et al., 2022), aspects of working memory and decision-making tasks (Hwang et al., 2019; Bellafard et al., 2024; Kuan et al., 2024), and conditioned versus extinguished sensory cues for fear memories (Grewe et al., 2017). In such investigations, the preference of each neuron for encoding one task-relevant state over another is assessed by comparing its responses to the two states in a statistically rigorous manner and assigning a selectivity label to the neuron: for instance, “State A”-selective versus “State B”-selective.

For such investigations, fluorescence imaging of neuronal calcium dynamics (“calcium imaging”) has emerged as a popular method to measure neural activity owing to its superior ability to access targeted (cell type- or projection-specific) neuronal subpopulations. These studies, however, have made diverse choices in the procedures and parameters used to infer neuronal selectivity. For instance, the type of neural data employed has varied: some studies use continuous Ca^2+^ traces (Grewe et al., 2017; Klaus et al., 2017; Gründemann et al., 2019), others use discrete Ca^2+^ events (Jimenez et al., 2018), and yet others use binarized Ca^2+^ events (Frost et al., 2021; Kuan et al., 2024). Whether or not neural data is binned beyond the frame rate of data acquisition has varied: no further binning (Klaus et al., 2017; Gründemann et al., 2019) versus temporally downsampled data (Grewe et al., 2017; Jimenez et al., 2018). The variable used to quantify the position of the animal in the behavioral arena, and therefore, the task-relevant state it occupies, has differed: some studies use the body-centroid as the measure (Jimenez et al., 2018; Courtin et al., 2022; Geva et al., 2023), whereas others use head-centroid (Fustiñana et al., 2021). The shuffling methods used to assess statistical significance of the selectivity of a neuron among behavioral states have also varied: some studies use a random permutation approach (randperm) to generate neuronal datasets under the null hypothesis of “no selectivity” (Jimenez et al., 2018; Hwang et al., 2019; Geva et al., 2023; Kuan et al., 2024) while others used a more structured, circular shifting approach (circshift; Gründemann et al., 2019; Frost et al., 2021; Fustiñana et al., 2021; Bellafard et al., 2024). Should different choices of parameter values influence the selectivity label assigned to a neuron, this would alter significantly interpretations about the encoding properties of the neurons and of the brain area. Despite this critical concern, an understanding of the impact of parameter choices on neuronal selectivity is currently lacking, as is an objective approach to assess the efficacy of any particular combination of parameter values and a recommendation of a preferred combination of parameter values for reliable inferences about neuronal selectivity. Bridging these gaps is essential for promoting robust interpretations about the neural encoding of task-relevant behavioral states and for connecting conclusions across studies.

Here, we address these gaps using as an experimental testbed, neural calcium dynamics imaged in the prefrontal cortex of freely behaving mice engaged in an exploration-avoidance task [elevated zero maze (EZM)]. We first employ an oft-used “selectivity index” (SI) procedure for assessing the preference of individual neurons to task states. Based on a large dataset of neurons imaged from eight animals, we find that a number of key parameters do affect significantly the selectivity label assigned to individual neurons. These include the type of neural data used, the type of behavioral data used, and the shuffling methods employed for statistical testing. Then, starting from first principles, we develop a novel approach to assess the “goodness” of any combination of parameter values using measures of “accuracy” in estimating neuronal selectivity and “robustness” (or consistency) in doing so. Using this approach, we identify an optimal combination of parameter values that produces the most reliable conclusions about individual neuronal selectivity. We validate this conclusion using a bootstrap approach to account for variability in the dataset and estimate its applicability to novel data using a cross-validation approach. Additionally, we demonstrate the generality of this combination of parameter values by employing a different (regression-based) procedure for assessing the preference of individual neurons to task states. This optimal combination utilizes calcium events convolved with a 2 s exponential decay filter, head-centric animal position data, 50 ms binning of data, animal-controlled dataset sizes for task states, and randperm shuffling for statistical testing.

Taken together, results from this work establish a standardized approach to reliably quantify, using calcium imaging, how individual neurons encode distinct task states.

## Materials and Methods

### Animals

The experiments were conducted using eight C57BL/6 mice obtained from The Jackson Laboratory. The mice were allowed to acclimatize to the animal care facility for at least a week, under a 12 h light cycle (7 A.M.–7 P.M.) with constant temperature (22°C) and humidity (40%), before starting any testing procedures. Food and water were provided *ad libitum* throughout the experiments. Experiments occurred during the standard light cycle. All protocols and animal care were in accordance with NIH guidelines for care and use of laboratory animals and approved by the Johns Hopkins University Institutional Animal Care and Use Committee.

### Surgery

We performed surgery and virus injections on four male and four female mice at 10–12 weeks of age. Briefly, the mouse was allowed to acclimatize to the surgery room for 30 min upon which it was placed in an anesthetic chamber (3% isoflurane, 1.5 O_2_ level) for 5 min. The mouse was then secured in a stereotactic frame (Kopf Instruments). Temperature was maintained at 36.8°C by placing a heating pad under the mouse's body. Craniotomy was made at the appropriate stereotaxic coordinates to target the mPFC. Two injections of AAV1/5.CAMKII.GCaMP6f.WPRE.SV40 (350 nl each) were made using a 0.5 ml micro syringe and motorized pump (Harvard Apparatus); syringe needle was lowered slowly at the rate of 200 µm/min into the mPFC region of the mouse brain (*y*_1_ = 1.86 mm AP, *x*_1_ = 0.25 mm ML, *z*_1_ = −2.80 mm DV, *y*_2_ = 1.34 mm AP, *x*_2_ = 0.25 mm ML, *z*_2_ = −2.80 mm DV relative to bregma). Immediately following virus injections, a gradient index (GRIN) lens (1 mm W, 4 mm L, Inscopix) was implanted into the mPFC (*y*_lens_ = 1.60 mm, *x*_lens_ = 0.25 mm, *z*_lens_ = 2.50 mm). The microendoscopic lens was also lowered at the rate of 200 µm/min into the brain and secured using Metabond. Upon completion of surgical procedures, the animal was given intraperitoneal injections of meloxicam (0.1 ml) and dexamethasone (0.1 ml), allowed to rest in a cage under a heat lamp until recovery, and then returned to the animal facility. Meloxicam (0.1 ml) was injected once daily for the next 3 d. Following 4–6 weeks to allow for virus expression, the expression level was checked by connecting the lens to the microscope (nVista HD, Inscopix). Upon sufficient expression of the virus, the mouse was ready for data collection experiments.

### Behavioral procedures

Experimental animals were single-housed in behaviorally enriched home cages on a 12 h light cycle. The elevated zero maze (EZM) constituted a circular platform raised above ground level (6.1 cm W, 40 cm inner diameter, 72.4 cm above ground) and divided into four quadrants. Two opposite quadrants were enclosed by walls of 20.3 cm (“closed arms”), while the other two arms did not have any walls (“open arms”). A camera was centered above the maze apparatus to record mouse behavior. The apparatus was placed within an area surrounded by a thick black curtain, lights were dimmed. Following a 30 min acclimation to the experimental room, animals were placed in a designated open arm of the EZM, and their freely moving behavior was recorded for 20 min. An automated behavioral monitoring system (EthoVision, Noldus v11.5; frame rate, 18.96 Hz) was used to track the animal’s motion trajectories from the recorded videos. The animal was returned to its home cage and the animal facility at the end of the experiment.

### Data collection

Neural activity was recorded using cellular-resolution fluorescence calcium imaging of ensembles (50–120 neurons) in the mPFC during EZM behavior. The change in fluorescence and the behavioral video were simultaneously recorded. The two were synchronized to ensure the same start and end time by using an Arduino-based trigger system: the Arduino-generated digital TTL pulses were simultaneously sent to both systems to trigger the start and mark the end of each recording session, ensuring temporal alignment. The calcium imaging data were preprocessed by the Inscopix Data Preprocessing Software (IDPS), outputting the neurons identified and the neural traces and events data.

### Preprocessing of calcium imaging data

Raw movie data were preprocessed using the IDPS. The movies were cropped, spatially downsampled by a factor of 4, bandpass filtered (low cutoff = 0.005, high cutoff = 0.5), and motion corrected. After preprocessing, PCA/ICA analysis was computed to select for individual neuronal components (see Inscopix IDPS manual for further details). Fluorescence traces for each component were extracted by the software as the average pixel intensity within the normalized ROI projected along the filtered and motion-corrected 20 Hz raw fluorescence movie. We manually inspected the traces, and those that failed to pass quality criteria (sharp peaks with an action potential-like decay across 1–4 s time period) were excluded. We typically retained 70–90% of the components after applying the above criteria. Of the cells that we accepted, we used the traces and events data (events threshold factor = 4; events smallest decay time = 0.2) outputs from the IDPS software for further analysis. Data were imported into MATLAB for analysis using custom-written software.

To derive the convolved version of the discrete Ca events, we convolved the events with either a 2 s or a 4 s exponential decay filter. To eliminate the low-frequency drift underlying the Ca traces, we applied a low-pass filter to the Ca traces and subtracted the slow drift component from the original data. Subsequently, we rectified the low-pass-subtracted Ca traces by setting all negative values to zero. For all analyses of the Ca traces, we utilized the rectified low-pass-subtracted Ca traces.

### Determination of neuronal selectivity for task-relevant states

Only neurons with >10 Ca^+^ events were selected for analysis. A total of 692 neurons from eight animals were eventually included in the analysis. To determine the selectivity level of each neuron, we first calculated its selectivity index (SI). The open-arm (OA) activity was defined as the average Ca^+^ activity of the neuron during the time when the animal stayed in the open arm. We used either the matched or the nonmatched method to determine the closed-arm (CA) activity. In the nonmatched method, the average Ca^+^ activity of the neuron during the time when the animal stayed in the closed arm was used. The matched method randomly selected and matched closed-arm bouts that had the same duration as the open-arm bouts 1,000 times. Each time, we calculated CA activity as the average Ca^+^ activity of the neuron during closed-arm bouts and then took the average CA activity of these 1,000 values. SI was calculated as follows:

$$\mathrm{SI}=\frac{\mathrm{avg}(\mathrm{OA})-\mathrm{avg}(\mathrm{CA})}{\mathrm{avg}(\mathrm{OA})+\mathrm{avg}(\mathrm{CA})}.$$

After obtaining the SI, a null distribution of SI was generated for each neuron. The null distribution was obtained by shuffling the Ca^+^ activity for 1,000 iterations. We set *p* = 0.05 as our decision threshold, so the neurons with actual SI >97.5th of the null distribution were classified as OA-selective, smaller than 2.5th of the null distribution as CA-selective, and the rest as nonselective.

In addition to the raw Ca events data, we extended the application of the SI calculation and selectivity classification procedure to the convolved Ca events data and Ca traces. The methodologies remained consistent with those described above, with the exception that the Ca events were substituted with convolved Ca events or Ca traces. For the letter two cases, we utilized the area under the curve (AUC) as the metric for neural activity.

### Shuffling methods

We explored four distinct shuffling methods to obtain the SI null distribution for statistical testing of the selectivity of each neuron. In the “randperm” method, we shuffled the Ca activity in time. When applying randperm to convolved Ca events, we initially shuffled the discrete Ca events and then applied the calcium-transient filter to the shuffled events to obtain the shuffled convolved events. In the “partial events-chunking” method (partial ECM), we first identified the time points at which a calcium (Ca) event occurred. We then defined each event chunk as the neural signal for *x* (*x* = 2 for 2S convolved Ca events, *x* = 4 for 4S convolved Ca events, *x* = 3 for Ca traces and Ca events) seconds following the event, which corresponds to the approximate time needed for a Ca event to decay. These event chunks were randomly shuffled, and the time points outside of these chunks were set to zero. The “full events-chunking” method (full ECM) resembled the partial ECM, except that a chunk of event was defined as the neural signal between the occurrence of an event and the occurrence of the next event. In the “circshift” method, we shifted the Ca activity in a certain direction by a random duration.

### Calculation of label consistency across pairs of parameter combinations (“settings”)

We evaluated 128 unique combinations of parameter values for characterizing individual neuron selectivity. These combinations were derived from five parameter dimensions: behavioral data type (2 levels), neural data type (4 levels), binning level (2 levels), matching level (2 levels), and shuffling method (4 levels; 128 combinations = 2 × 4 × 2 × 2 × 4). However, since the randperm shuffling method does not apply lawfully to raw traces, 8 combinations were excluded, resulting in 120 valid parameter combinations.

The label consistency between two given parameter combinations was defined as the percentage of neurons assigned to the same selectivity labels by both combinations. To calculate the 95% bootstrapped confidence interval (CI) for label consistency, we resampled neurons with replacement 1,000 times. For each bootstrapped sample, we computed label consistency, and the 95% CI was derived from the resulting bootstrap distribution of label consistencies. To determine the degree of each parameter’s effect on label assignment, we compared the lower bound of the 95% CI of label consistency to thresholds of 0.9 and 0.8 (Fig. 3*B–G*). If the lower bound was >0.9 (i.e., the consistency value was significantly >0.9), the parameter was considered to have a small effect, and the entry was shaded red. If the lower bound was <0.9 but >0.8 (i.e., the consistency value was not significantly >0.9 but significantly >0.8), the parameter was considered to have a medium effect, and the entry was shaded orange. If the lower bound was <0.8 (i.e., the consistency value was not significantly >0.8), the parameter was considered to have a large effect, and the entry was shaded yellow.

### True SI and null SD consistency analysis

To compare whether the computed selectivity index (SI) or the standard deviation of the null SI distribution (null SD) from two parameter combinations differed significantly, we used robust linear regression (*robustfit* in MATLAB) to fit a line to the true SI or null SD values of neurons produced by both parameter settings. This function uses an iteratively reweighted least-squares algorithm, which is less sensitive to outliers than ordinary least-squares regression.

We assessed whether the slope of the regression line was significantly different from 1 at a significance level of 0.01. A slope was deemed significantly different from 1 if the 99% confidence interval (CI) of its estimate did not include 1. To calculate the 99% CI, we performed 1,000 bootstrap iterations, fitting a regression model to each bootstrapped sample. The 99% CI was obtained from the resulting bootstrap distribution of slope estimates. A 99% CI was used (as opposed to the 95% CI applied for thresholding label consistency) as a heuristic means to apply corrections for multiple comparisons at the 95% confidence level; this effectively provided a more conservative estimate of the number of entries with true SI or null SD slopes significantly different from 1, identifying these entries as problematic.

### Boundary effect analysis

We defined near-boundary regions as areas within one head-to-body center length on either side of the four OA–CA boundary lines. This distance was estimated to be 4.04 cm—the median head-to-body center length measured across all frames of behavioral sessions from four randomly selected mice (out of the eight used in this study; Extended Data Fig. 3-1*H*). We, then, excluded all time points for which the mouse’s body center lay within these near-boundary regions (Extended Data Fig. 3-1*H*). The head-to-body center length was chosen as the exclusion criterion because it represents the minimum spatial scale at which the head and body could occupy different arms (OA or CA), creating ambiguous states. Removing data points within this distance from the boundary therefore avoids ill-defined behavioral states during transitions between OA and CA.

We then recalculated the selectivity labels of neurons under all parameter combinations using the boundary-excluded data. Based on the new label assignments, we computed label consistency across the 120 parameter settings and regenerated the 120 × 120 thresholded label consistency matrix as in Figure 3*B* (Extended Data Fig. 3-1*I*).

### Identification of template neurons for assessing the accuracy of parameter combinations

Template neurons were defined as those exhibiting distinctive firing patterns during the animal’s occupancy of the OA and CA behavioral zones. To qualify as a template OA/CA-selective neuron, the normalized difference in average firing rates between the OA and CA arms had to exceed 0.85. To qualify as a template nonselective neuron, the normalized difference in average firing rates between the OA and CA arms must be 0.05 or less.

### Calculation of robustness of parameter combinations

This is described in detail in Figure 4.

### Validation of the optimal parameter combinations using a bootstrapping procedure

To validate the optimal parameter combinations with respect to the variability in the dataset, we employed a bootstrapping procedure. A total of 500 bootstrap samples were generated, each matched in size (692 neurons) and composition to the original dataset. This included the same number of template neurons for each type and nontemplate neurons: 33 template neurons (7 OA-selective, 13 CA-selective, and 13 nonselective) and 659 nontemplate neurons.

For each bootstrap iteration, accuracy and robustness were calculated for each of the 120 parameter settings. Accuracy was determined using the bootstrapped template neurons, while robustness was obtained using the entire bootstrapped sample, including both template and nontemplate neurons. The optimal parameter setting for each iteration was identified as the one yielding the highest accuracy and robustness. Results across the 500 iterations were used to identify the most frequently occurring optimal setting.

### Estimation of the applicability of the optimal parameter combinations to unseen data using holdout cross-validation

To evaluate the generalizability of parameter settings or, in other words, the generalization error, we performed a holdout cross-validation analysis. In each iteration, the dataset was randomly partitioned into a training set (60% of the data) and a testing set (40% of the data). A total of 200 training–testing pairs were generated. Both template and nontemplate neurons were proportionally resampled so that each partition contained approximately 60% (training) and 40% (testing) of each neuron type. As a result, each training subset contained 20 template neurons (4 OA-selective, 8 CA-selective, and 8 nonselective) and 395 nontemplate neurons, while each testing subset contained 13 template neurons (3 OA-selective, 5 CA-selective, and 5 nonselective) and 264 nontemplate neurons. For each partition, accuracy and robustness were computed for each of the 120 parameter settings using first the training set and then the testing subset. As before, accuracy was determined using the sampled template neurons, while robustness was obtained using the entire sample (template and nontemplate neurons combined). Parameter settings were then ranked within each subset based on their accuracy and robustness scores. Additionally, to go beyond the identity of the first- and second-optimal settings in assessing generalizability, we quantified how well the full ranking of parameter settings was preserved between training and testing subsets. Specifically, for each partition, we computed the Spearman’s rank correlation coefficient (*ρ*) between the training-derived and testing-derived rankings of all parameter settings. The distribution of *ρ* across 200 iterations was summarized, and the median *ρ* was used as a measure of the overall generalizability of rankings from training to testing data.

### Generality of the optimal parameter combinations in a GLM framework

To assess the generality of the optimal parameter combinations identified using the traditional selectivity index (SI) “approach” of determining individual neuronal encoding preferences, we used the generalized linear modeling (GLM) “approach.” Specifically, for each neuron, we modeled its instantaneous firing activity at each time point as a function of a binary behavioral regressor indicating whether the animal was occupying the open arm or not at that time point; no history terms (i.e., temporal lags) were included in the regression models (Pinto and Dan, 2015; Chen et al., 2016).

Let *Y_t_* denote the neural activity at time *t*, and let *X_t_* denote whether the animal was in the open arm (*X_t_* = 1) or the closed arm (*X_t_* = 0) at time *t*. Under the linear model with an identity link function, the model was as follows:

$$Y_{t}=\beta_{0}+\beta_{1}*\;X_{t}+\varepsilon_{t}\;\mathrm{where}\;\varepsilon_{t}∼N(0,\sigma^{2}).$$

Under the Poisson GLM with a log link function, the model was as follows:

$$Y_{t}∼\mathrm{Poisson}(\lambda_{t})\;\mathrm{where}\;\log(\lambda_{t})=\beta_{0}+\beta_{1}*\;X_{t}.$$

The resulting regression coefficient (*β*_1_ value) was used to assess neuronal selectivity. A neuron was classified as nonselective if its *β*_1_ value was not statistically significant different from zero at a threshold of *p* = 0.05. Among significant results, a positive *β*_1_ indicated selectivity for the open arm, while a negative *β*_1_ indicated selectivity for the closed arm.

To assess the statistical significance of each *β*_1_ coefficient, we employed a nonparametric permutation test. Specifically, we shuffled the behavioral regressor (*X_t_*) and refit a GLM for the shuffled dataset. We repeated this procedure 100 times and generated a null distribution of *β*_1_ values. Empirical *p*-values were calculated as the proportion of shuffled *β*_1_s whose absolute values exceeded the absolute value of the observed coefficient.

We tested the generality of the top two parameter settings identified through the SI-based analysis for the GLM-based analyses. Each of the two optimal parameter settings (SI approach) was combined with either an identity link function (corresponding to linear modeling, LM) or a log link function (corresponding to Poisson GLM), resulting in four unique parameter combinations (Fig. 6*B*). To evaluate whether these parameter settings were effective within the GLM framework (and to determine which link function was more appropriate), we quantified label consistency, defined as the percentage of neurons assigned the same selectivity label (OA-selective, CA-selective, or nonselective) by both the SI and GLM approaches.

## Results

### Study design

**Figure 1.**
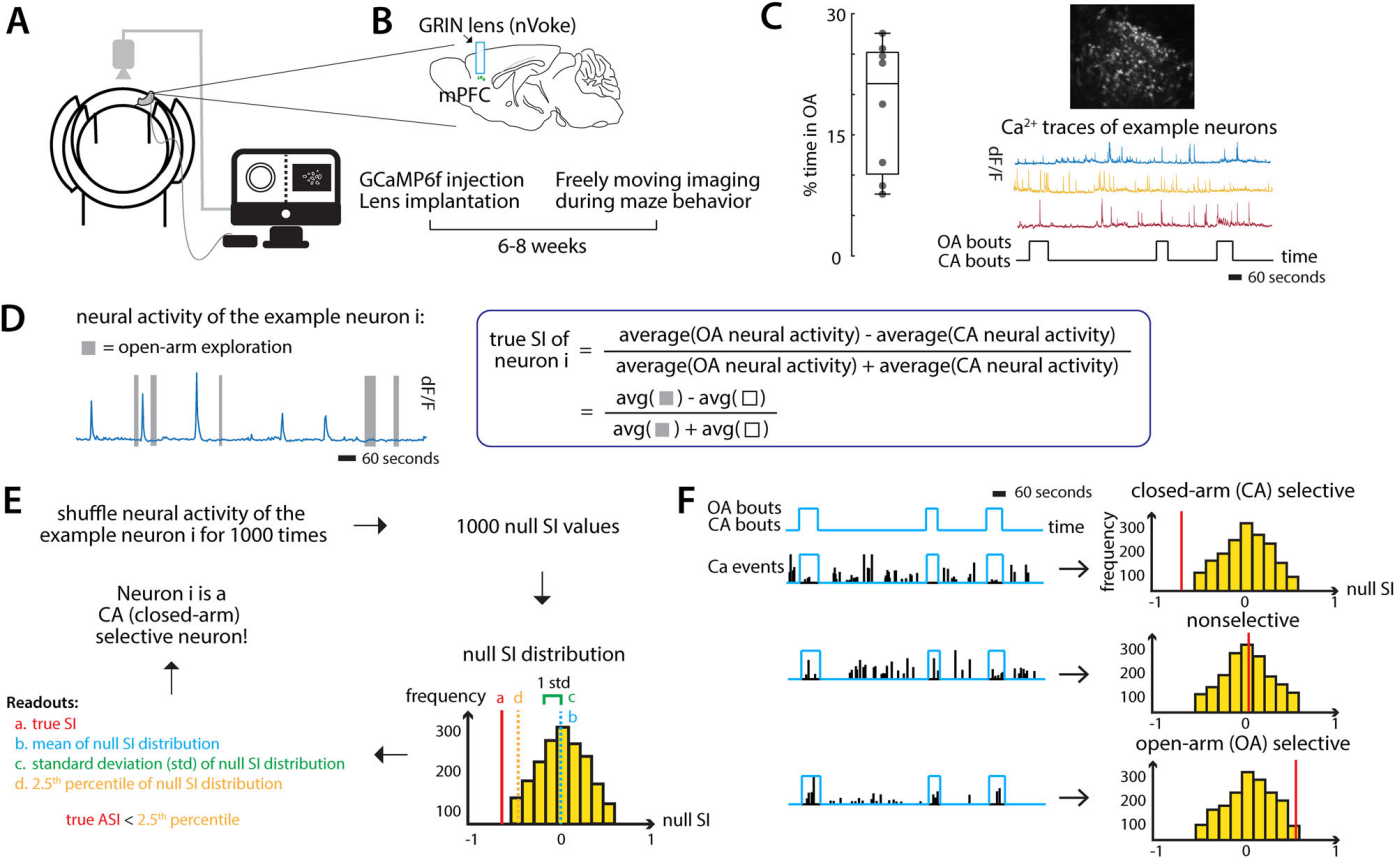
Study design. ***A***–***C***, Experimental setup. ***A,B***, Schematic of the experimental setup: AAV1/5 CaMKII-GCaMP6f virus is injected into the mPFC, and a GRIN lens is implanted above the injection site. After waiting 6–8 weeks for virus expression, the animal is placed on the elevated zero maze where its behavior and the calcium activity of simultaneously imaged mPFC neurons are recorded. ***C***, Left, Box plot showing the percentage of time spent by mice in the open arm; each point represents an individual mouse (*n* = 8). Right, Imaged mPFC neurons expressing GCaMP6f from an example animal (top) and calcium traces of example neurons (bottom) extracted from the recorded video of a 20 min session. The time spent in open arms and closed arms is also shown. ***D–F***, Characterizing the selectivity of individual neurons to open-arm versus closed-arm using a selectivity index. ***D***, Left, Calcium transients of an example neuron that fired preferentially in the closed arm. The time spent in open arms is shown by gray bars. Right, The formula for calculating the true selectivity index (SI) of an individual neuron. ***E***, Steps for determining the selectivity of an individual neuron. ***F***, Left, Calcium events from the three example neurons aligned to arm occupancy: open-arm selective (top row), closed-arm selective (middle), and nonselective (bottom). Right, SI null distribution for each of these neurons obtained by computing SI values from shuffled data (*n* = 1,000 iterations; Materials and Methods). Red line: true SI value of the neuron.

To investigate the dependence of inferred neuronal selectivity for task states on parameter values in the inference process, we chose mice as our animal model, and studied spontaneous navigation by freely behaving mice in the elevated zero maze (EZM; Shepherd et al., 1994; Fig. 1*B*). The EZM consists of two distinct spatial zones—exposed, “open-arm” segments representing potentially unsafe zones, and enclosed, “closed-arm” segments representing potentially safe zones (Fig. 1*B*). Navigation in the EZM is considered to be governed by an ongoing resolution of the conflict between two innate drives: the drive to avoid unsafe spaces and the drive to explore novel spaces. As a result, EZM navigation can be described in terms of two task-relevant states: occupancy of open-arm segments, during which the exploratory drive exceeds the aversive drive, and occupancy of closed-arm segments, during which the opposite is true (Montgomery, 1955; Shepherd et al., 1994). Behavioral assays involving such unconditioned navigation of potentially safe versus unsafe zones have been used extensively in the literature to study the neural basis of affective decision-making (Adhikari, 2014; Calhoon and Tye, 2015; La-Vu et al., 2020).

In mice engaged in the EZM assay, we investigated neural representations in the medial prefrontal cortex (mPFC; Fig. 1*C*), a brain area heavily implicated in affective decision-making (Adhikari et al., 2011; Calhoon and Tye, 2015; Tovote et al., 2015; Malezieux et al., 2023). Specifically, neural calcium dynamics in the mPFC were visualized by expressing virally the genetically encoded fluorescent calcium indicator, GCaMP6f (Chen et al., 2013; Materials and Methods) and by performing cellular-resolution calcium imaging with a GRIN lens using endoscopic miniscopes (Resendez et al., 2016; nVista HD, Inscopix; Fig. 1*C,D*). Our dataset from this study design consisted of 692 individual mPFC neurons imaged from eight mice freely exploring the EZM.

To characterize the selectivity of each neuron to the two task-relevant states in the EZM, namely, open-arm occupancy versus closed-arm occupancy, we computed a standard modulation index used frequently in the literature (Herrero et al., 2008; Jimenez et al., 2018; Gründemann et al., 2019). Referred to here, as the selectivity index (SI), it is defined as the difference between the average neural responsiveness during open-arm exploration versus closed-arm exploration, divided by their sum (Materials and Methods; Fig. 1*D*). The values of SI range from −1 to +1 and indicate the degree to which a neuron is preferentially active during open-arm occupancy (positive SI values) versus closed-arm occupancy (negative SI values). Specifically, a neuron is said to be selective for one of the states if its computed SI value is significantly different from a null distribution of SI values obtained by shuffling its open-arm and closed-arm neural responses (Fig. 1*E*; Materials and Methods). The computed SI values and null SI distributions of three example mPFC neurons that are inferred to be, respectively, CA-selective, nonselective, and OA-selective, are illustrated in Figure 1*F*.

### Key parameters relevant to characterizing neuronal selectivity for task states

Characterizing the selectivity of individual neurons to task states necessitates a priori choices of various parameters along the experimental and analysis pipeline, which consists of three major stages (Fig. 3*A*). Here, we identify the key parameters in each stage, discuss which ones merit inclusion for investigation, and why.

**Figure 2.**
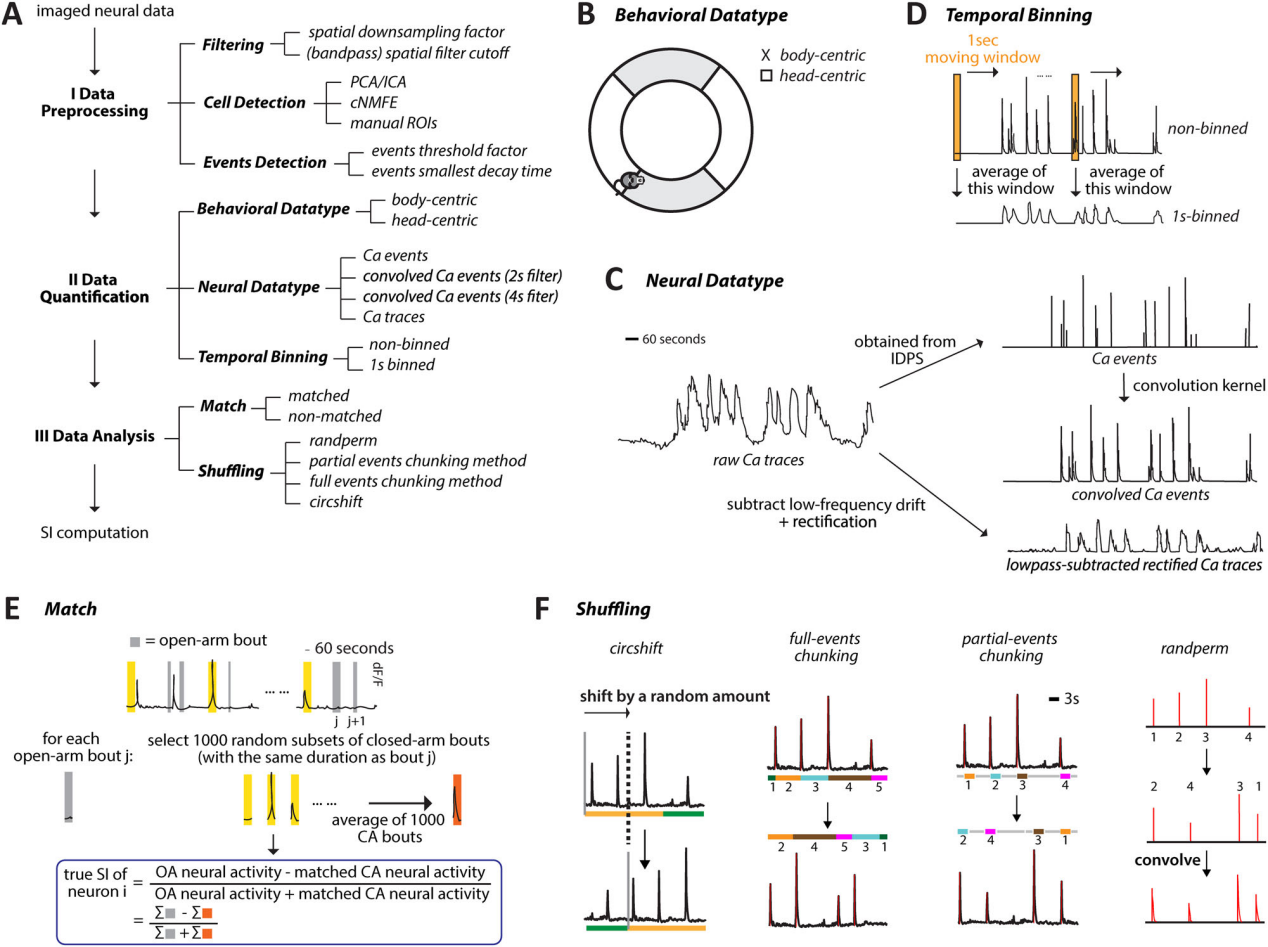
Description of key parameters included in the investigation. ***A***, Key parameters involved in each of the three major data processing stages from data collection to assessment of selectivity. Effects of preprocessing (Step I) on calcium data are shown in Extended Data Figure 2-1. ***B***, Using a single coordinate to characterize the position of the mouse in the maze: head-centroid position versus body-centroid position. ***C***, Nonbinned calcium transients (top) versus 1 s-binned calcium transients (bottom). The 1 s-binned calcium transients were obtained by averaging values in a moving 1 s window. ***D***, Illustration of the types of neural data investigated in the current study. Raw Ca traces and Ca events were preprocessed and output directly from IDPS. Convolved Ca events were obtained by applying a convolution kernel to the Ca events. Ca traces analyzed in the current study referred to the low-pass-subtracted rectified Ca traces, which were obtained by subtracting the low-pass component from the raw Ca traces and rectifying them. ***E***, Illustration of the number-matched method. The time spent in open arms is shown by the gray bars. The yellow bars refer to random subsets of closed-arm bouts. ***F***, Representations of the procedures of the four shuffling methods.

The first, “preprocessing,” stage involves (Fig. 2*A*): (1) spatial filtering of raw fluorescence videos obtained from the microscope, followed by (2) identification of individual cells (and their DF/F calcium traces) from these videos, followed by (3) the detection of calcium events from the DF/F trace of each cell. The parameter choices involved in all three steps of this first stage do not significantly affect the eventual computation of neuronal selectivity, as described next. (1) For the “spatial smoothing” step (Fig. 2*A*), a range of standard filters are used commonly used in the literature, differing in the spatial downsampling factor and the low/high filter cutoff values. Different filter specifications can quantitatively affect the DF/F traces of calcium activity extracted from the videos, thereby potentially affecting the eventual computation of the neuronal selectivity index. In practice, however, we found that the calcium traces extracted using a range of commonly used spatial filter parameter values were highly correlated with one another (Extended Data Fig. 2-1*C*), revealing a minimal potential impact of typical spatial filtering rules on the eventual computation of selectivity. (2) For the “cell identification” step (Fig. 2*A*), the commonly used approaches are manual identification, PCA/ICA analysis (followed by manual verification), or cNMFE analysis (followed by manual verification). Although these different approaches can impact the number and identity of detected neurons, they do not, per se, impact the extracted calcium dynamics of any identified neuron, and therefore, the computation of its selectivity. Finally, (3) for the step of “detection of rapid calcium transients” (or “calcium events”) from the DF/F traces of individual cells (Fig. 2*A*), a range of standard procedures are used commonly, differing in the event threshold factor (the threshold below which a spike is not considered a rising event) and in the event decay time factor (the minimum value of the mean lifetime of the decay of a spike). The number of events detected in a trace can depend on the rules used, thereby potentially impacting the computation of selectivity. In practice, however, we found that the time courses of detected events were highly correlated among the commonly used procedures (Extended Data Fig. 2-1*D*), revealing a minimal potential impact of typical event detection rules on the eventual computation of selectivity. Consequently, the parameters involved in this first, preprocessing, stage of the pipeline were fixed to typical values for subsequent investigation (see Materials and Methods).

10.1523/ENEURO.0378-25.2025.f2-1 Figure 2-1 Effects of preprocessing on calcium data. **(A)** Key parameters in the data preprocessing stage and the standard range of choices for each parameter. **(B)** Procedure for inferring the overall effect of each preprocessing parameter on the output calcium data. **(C)** Median correlation coefficient between traces produced by methods using different parameters for spatial smoothing across all neurons. **(D)** Median correlation coefficient between events produced by methods using different parameters for event detection across all neurons. Download Figure 2-1, TIF file.

The second, “quantification,” stage involves (Fig. 2*A*): (1) quantifying the behavioral position of the mouse in the maze, (2) quantifying the neural activity, and (3) temporally binning the behavioral as well as neural data. (1) For quantifying “mouse position” (Fig. 2*B*), the commonly used metrics are “body” position (estimated centroid of the body of the mouse in each video frame) or “head” position (estimated center of the head in each frame). Given that mice can exhibit behaviors involving head movements that are not captured by body position alone, such as exploratory head extensions into the open arm with the body still in the closed arm, these two variables can produce differing assessments of the task-relevant zone occupied by the mouse at any instant (and thereby, potentially affect the eventual computation of neuronal selectivity). We included them as our two “behavioral datatype” levels for subsequent investigation. (2) For quantifying “neural calcium dynamics” (Fig. 2*C*), we employed four metrics. We included the two commonly used metrics of area under the curve of continuous calcium traces and the number of discrete calcium transients (events) detected from these raw traces. Continuous traces provide the most “complete” description of calcium dynamics, but because of the slow (seconds-long) decay in calcium kinetics, calcium fluorescence changes that are initiated just before the animal executes a state transition can leak into the posttransitional state resulting in potential misattributions (overestimation) of area-under-the-curve activity for the second state. In contrast, discrete calcium events are much less subject to such misattribution errors but are more susceptible to nonlinear effects in the spike-to-calcium transformation. In addition, we introduced two other metrics: discrete Ca^2+^ events convolved with a single exponential decay kernel of time constant 2 or 4 s, respectively (to mimic fast vs slow GCaMP6f vs 6 s kinetics). Both these convolved traces retain the advantage of greater statistical power that is offered by continuous traces (over discrete event traces; Cunningham and Yu, 2014), while minimizing the impacts of noise and drift often found in raw calcium traces. In total, we had four different “neural datatype” levels for subsequent investigation. (3) Finally, for “temporal binning” of both the calcium and behavioral data (Fig. 2*C*), the commonly used choices in the literature range from no binning (beyond the original 20 Hz sampling rate of miniscope data, i.e., 50 ms bins) to binning data into 1 s bins. We included these two extremes (50 ms vs 1 s bins) as our two binning levels for subsequent investigation.

The third, “analysis,” stage is the one in which the encoding preference of each neuron is computed. It involves choices with respect to two key steps (Fig. 2*A*): (1) balancing (or not) the number of data samples between task states and (2) the shuffling method employed to obtain the null distribution for statistically rigorous inference of neuronal selectivity. (1) With respect to data balancing (or matching), because the amount of time mice spend in different task states cannot be guaranteed to be equal in tasks involving spontaneous state transitions, an imbalance in the number of data points available for different task states is commonplace. For instance, in our task, mice tend to spend a greater fraction of time in the potentially safe zone (closed arm) compared with the aversive zone (open arm). Such an imbalance could potentially introduce biases in the assessment of neuronal selectivity. To address this issue, we decided to compare the impact of matching exactly the number of closed-arm and open-arm data samples (through random subsampling of closed-arm data), versus not matching them (Materials and Methods; Fig. 2*E*). (2) With respect to data shuffling for null distribution generation, we explored four different methods (Fig. 2*F*). “randperm” and “circshift” are the most commonly used shuffling methods in the literature, referring, respectively, to randomly rearranging neural data samples in time, versus to shifting neural time-course with respect to the behavioral time-course by a random duration. The former method fully randomizes the association between neural and behavioral data, whereas the latter injects a smaller degree of randomness into this association while retaining local (temporal) structure in the neural data. To better explore the tradeoff between fully randomizing the data versus randomizing while retaining local temporal structure, we introduced two additional shuffling methods that we refer to as “partial events-chunking” (partial EC) and “full events-chunking” (full EC) methods. Both maintained the integrity of “local” calcium signals, i.e., signals within chunks of time defined with respect to the timing of calcium events. In the full EC method, we defined an event chunk as the neural signal from one event until the next and then shuffled these chunks in time. In the partial EC method, we modified this idea by defining an event chunk as the duration from one calcium event to *x* seconds after it (*x* = 2 for 2 s convolved Ca events, *x* = 4 for 4 s convolved Ca events, *x* = 3 for Ca traces and Ca events) and then shuffled these chunks in time.

Taken together, three key parameters from the data quantification stage and two key parameters of the data analysis stage described above produced a total of 120 unique combinations of parameter levels for characterizing the selectivity of individual neurons (120 = 2 behavioral datatypes × 4 neural activity datatypes × 2 binning widths × 2 matching levels × 4 shuffling methods—8 invalid combinations; see Materials and Methods). Henceforth, each unique combination of parameter levels will be referred to as a parameter “setting” (with 120 distinct parameter settings).

### Do parameter choices matter, and if so, how?

To investigate whether different parameter settings have an impact on the inferred selectivity of individual neurons for task-relevant states, we asked whether they altered the selectivity label assigned to each neuron—namely, OA-selective, CA-selective, or nonselective. Our reasoning was that any change in the assigned label would reveal a major error in characterizing the neuron's encoding preference arising simply due to a different combination of parameter levels used in the analysis pipeline.

**Figure 3.**
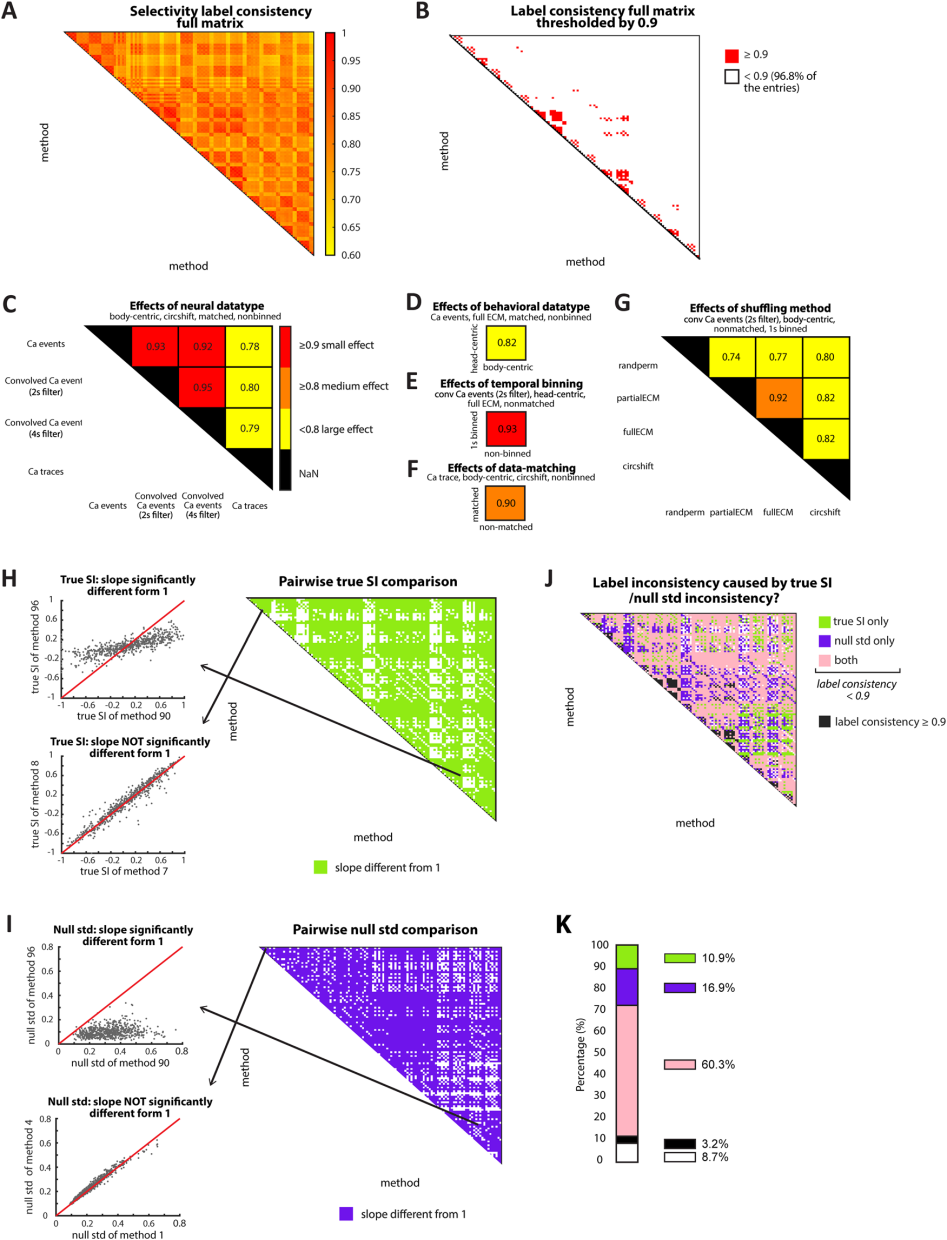
Effects of parameters on label consistency and mechanisms of inconsistency. ***A***, Pairwise label consistency matrix. A 120 × 120 matrix visualizing the effect of parameter settings on neuron selectivity labels (OA-selective, CA-selective, or nonselective). Label consistency: % of neurons assigned the same label by two parameter combinations. Note that randperm does not apply lawfully to raw traces, so eight combinations were excluded. We only show the top diagonal part of the matrix because it is symmetric. Bootstrap confidence intervals (CIs) are not shown here but are incorporated in ***B***. ***B***, Thresholded label consistency matrix (from ***A***). Red shading (small effect, consistency values significantly >0.9); significance computed using bootstrap confidence intervals (CIs) for each matrix entry (Materials and Methods). The label consistency matrix with a threshold value of 0.8 is shown in Extended Data Figure 3-1*A*. ***C–G***, Submatrices of parameter effects. Example pairwise label consistency submatrices illustrating the main effects of each parameter. Red shading (as in ***B***), orange shading (medium effect, consistency values <0.9 but significantly >0.8), and yellow shading (large effect, consistency values not significantly >0.8); see Materials and Methods. More example submatrices are shown in Extended Data Figure 3-1*B–F*. ***H***, Congruence matrix of computed SI between all pairs of parameter settings (120 × 120; right). Each entry represents whether the line fit to the scatter plot of SI values from that pair deviated significantly from 1 (bootstrap approach; Materials and Methods). Left, Two example best-fit lines, representing low (top) and high (bottom) true SI congruence. ***I***, Congruence matrix of null SD values, similar to ***H***. ***J***, Matrix showing congruence of computed SI and null SD: merge of ***H*** and ***I***, filtered by ***B***. Black indicates parameter pairs with label consistency significantly >0.9. For the rest, green indicates low congruence of computed SI values, purple indicates low congruence of null SD values, and pink indicates low congruence for both metrics (see Materials and Methods). ***K***, Percentage of each type of entry from the matrix shown in ***J***.

To this end, we quantified the “label consistency” between each pair of parameter settings as the percentage of neurons assigned the same labels by both settings. This produced a 120 × 120 matrix of (pairwise) label consistency values (Fig. 3*A*). Examining these values together with their 95% bootstrap confidence intervals (Materials and Methods) revealed surprisingly poor consistency in assigned neuronal selectivity labels between most pairs of parameter settings. Indeed, 96.8% of the entries were lower than 0.9 consistency (Fig. 3*B*; see Materials and Methods), and nearly three-fourths (74.3%) of the entries were lower than an even more permissive cutoff value of 0.8 consistency (Extended Data Fig. 3-1*A*; see Materials and Methods).

10.1523/ENEURO.0378-25.2025.f3-1 Figure 3-1 **Effects of parameters on label consistency, and mechanisms of inconsistency.** **(A)** Thresholded label consistency matrix (from Fig. 3A). Orange shading (consistency values significantly greater than 0.8); significance computed using bootstrap confidence intervals (CIs) for each matrix entry (Methods). **(B1-F1)** Submatrices of parameter effects. Example pairwise label consistency submatrices illustrating the main effects of each parameter. Red shading (as in B), orange shading (medium effect, consistency values less than 0.9 but significantly greater than 0.8), and yellow shading (large effect, consistency values not significantly greater than 0.8); see Methods. **(B2-F2)** Pie charts summarizing parameter main effects and interaction effects between parameters. For C2-E2, pie charts visualize the distribution of consistency levels (red, orange, yellow) across all submatrices representing the parameter’s effect. For example, in the matched-nonmatched pie chart (E2), percentages of red/orange/yellow entries were calculated from 64 submatrices (squares) related to “effects of data-matching.” The dominance of orange entries indicates that matched and nonmatched generally had a medium effect across all conditions. Interaction effects. Pie charts also highlight interaction effects between parameters. In the absence of interactions, pie charts would display homogeneous colors (e.g., all red or orange). However, most pie charts demonstrate mixed colors, indicating significant interactions among parameters. For B2 and F2, the pie charts visualize the distribution of the main pattern (in B2, very low consistency between calcium traces and other neural data types, and high consistency among other pairs; in F2, very low consistency between randperm and partial ECM, and low consistency among other pairs) as well as variations within the main pattern. The presence of these variations indicates interaction effects between parameters. **(B3)** Example pairwise label consistency submatrices illustrating the interaction effects between parameters. **(G)** Matrix showing congruence of computed SI and null STD, filtered by Fig. 3B. Black indicates parameter pairs with label consistency not significantly greater than 0.9. For the rest, green indicates low congruence of computed SI values, purple indicates low congruence of null STD values, and pink indicates low congruence for both metrics (see Methods). **(H-I)** Boundary-zone exclusion. (H) (Left) Schematic illustrating the near-boundary regions relative to the four OA-CA boundary lines. (Right) Distribution of head-to-body center lengths (cm) across all frames from four mice. The median value was 4.04 cm. (I) Thresholded label consistency matrix (as in Fig. 3B) computed from boundary-excluded data. Red shading (consistency values significantly greater than 0.9); significance computed using bootstrap confidence intervals (CIs) for each matrix entry (Methods). Download Figure 3-1, TIF file.

Which parameters contributed most to inconsistencies in the inferred selectivity labels? To better understand this, we extracted submatrices of the consistency matrix to examine the effect of varying one parameter while keeping the others fixed. First, we examined the effect of varying “neural datatype,” with the other four parameters fixed to the following levels: behavioral datatype, “body-centric”; binning width, 50 ms”; shuffling method, “circshift”; and OA–CA data matching, “yes.” The corresponding submatrix revealed that although label consistency was high (>0.9) between “neural datatype” levels of “Ca events” and “Ca convolved events” (with either a 2 s/4 s filter; Fig. 3*C*, red shading), it was very low (<0.8) between the level of “Ca traces” and any of the other three neural datatype levels (Fig. 3*C*, yellow shading). This pattern (of very low consistencies in the last column of the submatrix) was observed for most combinations of levels of the remaining four parameters (Extended Data Fig. 3-1*B1*,*B2*; indicative of a main effect of “neural datatype”). Additional second-order patterns in these values were observed for specific combinations of the remaining four parameters (Extended Data Fig. 3-1*B2*,*B3*; indicative of interaction effects). Together, these results revealed that the “neural datatype” parameter, and specifically, its level “Ca traces,” exerted a large impact on the inferred selectivity labels.

Similarly, we examined the effect of varying each of the other two parameters from the “data quantification” stage, namely, “behavioral datatype” and “binning width.” We found that “behavioral datatype” exerted a large impact on the inferred selectivity label across combinations of levels of the remaining four parameters (Fig. 3*D*; Extended Data Fig. 3-1*C1*,*C2*; see Materials and Methods). “Binning width,” on the other hand, had only a small impact on the inferred selectivity labels (Fig. 3*E*; Extended Data Fig. 3-1*D1*,*D2*; see Materials and Methods). Then, we examined the effect of varying each of the two parameters from the “data analysis” stage. We found that the “OA–CA data matching” parameter had a medium impact on the inferred selectivity labels (Fig. 3*F*; Extended Data Fig. 3-1*E1*,*E2*; see Materials and Methods). The “shuffling method” parameter exerted a large impact on the inferred selectivity labels of neurons across combinations of levels of the other four parameters (Fig. 3*G*; Extended Data Fig. 3-1*F1*,*F2*; see Materials and Methods).

Together, these results revealed that parameter settings had a substantial effect on the inferred selectivity labels, with changes in the levels of most parameters producing medium to large effects.

How do these inconsistencies in label assignment arise? We recall that the selectivity label of a neuron depends both on its computed SI value and an assessment of whether this value is significantly different from the null distribution of SI values. We, therefore, asked if label inconsistencies between parameter settings were attributable to differences in the computed SI value, to the null distribution of SI or to both. To this end, we compared the computed SI values of neurons using one parameter setting against those computed using another setting (examples in Fig. 3*H*, left). If they differed significantly (slope of best-fit line different from 1; Materials and Methods), we concluded that a change in computed SI value was a major contributing factor to label inconsistency between these two settings. Across all pairs of parameter settings, this analysis yielded another 120 × 120 matrix—but this time, of congruence between computed SI values (Fig. 3*H*, right). Similarly, we generated a third 120 × 120 matrix of congruence of the SD values of the null SI distributions between pairs of parameter settings (Fig. 3*I*).

Using these two matrices, we assessed whether dissimilar true SI values, dissimilar null SD values, or both contributed to label inconsistency in the original matrix of Figure 3*B*. For the pairs of parameter settings with medium or large effects on consistency (Fig. 3*B*, white entries), we found that the majority showed significant changes in both computed SI and null SD (62.3%; Fig. 3*J,K*, pink shading), with some showing significant changes only in the computed SI value (11.3%; Fig. 3*J,K*, green shading) or only in null SD (17.5%; Fig. 3*J,K*, purple shading). Thus, the effects of parameter levels on selectivity labels can occur through different “mechanisms”—by affecting the true SI value, or the null SI distribution, or both.

One possible source of variability across parameter settings is the ambiguity of behavioral states at transitions between the open arm (OA) and closed arm (CA), when the mouse’s head and body can occupy different arms. To test the effect of this ambiguity in driving our results, we excluded near-boundary data points and reassessed label consistency across parameter combinations (Materials and Methods). Near-boundary regions were defined as areas within one head-to-body center length on either side of the four OA–CA boundary lines (median head-to-body center length of mice = 4.04 cm, calculated across all frames from four mice; Extended Data Fig. 3-1*H*; Materials and Methods). We then excluded all time points in which the mouse’s body center fell within these regions, recalculated selectivity labels of neurons for each of the parameter combinations, and regenerated the 120 × 120 thresholded label consistency matrix (as in Fig. 3*B*). The proportion of entries with label consistency significantly >0.9 increased from 3.2% with the full dataset to 4.8% with boundary-excluded data (Extended Data Fig. 3-1*I*). Although the exclusion of the near-boundary data led to a marginal improvement, variability across parameter combinations still remained extremely high. This suggests that the observed variability is not solely driven by potentially ambiguously defined behavioral states during arm transitions but rather reflects intrinsic variability due to different parameter settings.

Taken together, these results established the substantial and diverse impact of parameter choices along the pipeline, from calcium imaging data acquisition through analysis, on the characterization of the encoding properties of individual neurons for task-relevant states.

### What is the “optimal” parameter combination for assessment of neuronal selectivity?

Considering the extent of inconsistency in inferred neuronal selectivity introduced just by changing the levels of the key parameters, a critical question becomes: what choices of parameter levels “should” one use in order to make reliable inferences about the encoding properties of individual neurons? To answer this question, we drew inspiration from the well-established procedure of “model selection” in statistical analysis (Hastie et al., 2009). This involves assessing the accuracy (bias) as well as robustness (variance) of each “model” and then selecting the one with the highest accuracy and robustness as the “optimal” one. Here, the optimal parameter setting would correspond to the one offering the most reliable inferences.

**Figure 4.**
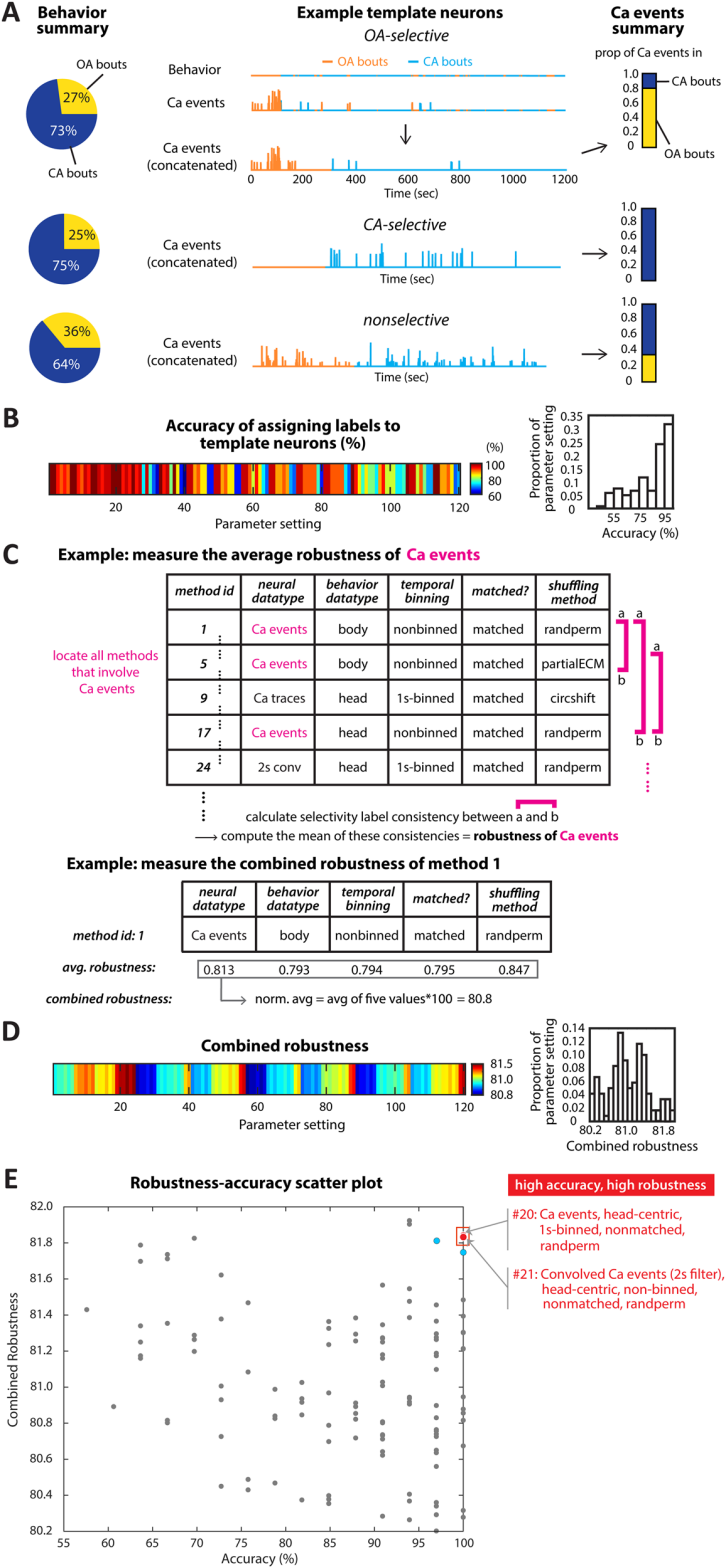
Accuracy and robustness analysis of parameter settings. ***A***, Template neuron examples. These neurons are from different mice. Left, Behavioral summary pie charts showing the percentage of time animals spent in OA and CA. Middle, Behavioral bouts and Ca events for an OA-selective template neuron are shown over time. To visualize patterns across behavioral bouts, OA/CA bouts and their associated Ca events were concatenated, ensuring that events occurring during OA (or CA) bouts remain grouped. Concatenated behavior bouts and Ca events are shown for all three example template neurons. Right, Proportion of Ca events occurring during OA versus CA bouts. ***B***, Parameter settings accuracy. The percentage of template neurons correctly assigned to their selectivity labels for each parameter setting. A breakdown of the accuracy of assigning labels to each type of template neurons (OA-selective, CA-selective, nonselective) is shown in Extended Data Figure 4-1. ***C***, Robustness calculation. Top, Procedure for calculating the average robustness of a specific parameter value, using calcium events as an example. Consistency values from Extended Data Figure 3*A* were used. Bottom, Calculation of combined robustness across all five parameter values. ***D***, Combined robustness. Robustness scores for all parameter settings. ***E***, Scatter plot of combined robustness versus accuracy. The red horizontal and vertical lines denote robustness (81.6) and accuracy (95) thresholds, respectively. Red dots: two (nearly overlapping) optimal parameter settings with the highest values of both accuracy and combined robustness. Blue dots: the next best settings behind the two optimal ones; they do not survive cross-validation (see text of Fig. 5).

First, we defined the “accuracy” of a parameter setting as its ability to assign selectivity labels to neurons “correctly.” Since the ground truth about neuronal labels is typically not known a priori, we developed a heuristic approach. We identified so-called “template” neurons as reference points for the evaluation of accuracy. They were defined as those exhibiting activity patterns during occupancy of the OA versus CA behavioral zones that were so distinctive that they could be used to incontrovertibly conclude their encoding preferences by simple visual inspection. Example template neurons that are, respectively, OA-selective, CA-selective, and nonselective, are shown in Figure 4*A*. A total of 33 template neurons (7 OA-selective, 13 CA-selective, and 13 nonselective) were identified from the full set of 692 neurons. Using these template neurons, we defined the accuracy of each parameter setting as the percentage of template neurons that it labeled correctly (Fig. 4*B*; Extended Data Fig. 4-1*A*).

10.1523/ENEURO.0378-25.2025.f4-1 Figure 4-1 **Accuracy and robustness analysis of parameter settings.** **(A)** Breakdown of Fig. 4B (parameter settings accuracy) by template neuron type. For each parameter setting, the percentage of OA-selective, CA-selective, and non-selective template neurons that were correctly assigned to their respective selectivity labels is shown. Download Figure 4-1, TIF file.

Next, we defined the “robustness” of a parameter setting to reflect the overall consistency in selectivity label inference associated with that parameter setting. This was based on the reasoning that label inconsistency (or inference variability) was undesirable and, therefore, that a parameter setting with greater consistency (i.e., higher robustness) was desirable. Specifically, we estimated the robustness of each parameter setting hierarchically: as a combination of the robustness of its constituent parameter levels. To this end, we first defined the “average robustness” of each “level” of a parameter as the mean of consistency values between all pairs of parameter settings that contained that level (Fig. 4*C*, top; consistency values from Fig. 3*A*; Materials and Methods). This metric captured quantitatively the extent to which the presence of that particular level of a parameter was associated with inconsistencies “under perturbations” to the levels of the other parameters. Consequently, in a choice between two levels of a parameter, the level with the lower value of average robustness is more likely to cause instability in the inferred selectivity and is, therefore, less optimal. Extending this reasoning from levels of a parameter to parameter settings as a whole, we defined the “combined robustness” of a parameter setting as the normalized combination of the average robustness of each of its five constituent levels (Fig. 4*C*, bottom; Fig. 4*D*; Materials and Methods).

With the accuracy and combined robustness of each parameter setting defined quantitatively, we constructed a 2D scatter plot of these two metrics in which each dot corresponded to one parameter setting (Fig. 4*E*). The most effective settings are, by definition, the ones with high values for accuracy (100%) and high values of combined robustness (>95th percentile). Two parameter settings displayed these desirable characteristics, and we designated them as “optimal.” One was #20 (acc = 100%, comb-robustness = 81.84), with the following constituent levels: neuronal activity datatype = Ca^2+^ events, behavioral datatype = head-centric data, temporal binning width = 1 s, OA–CA data matching = nonmatched; shuffling method = randperm. The other was #21 (acc = 100, comb-robst = 81.83), with the following constituent levels: neuronal activity datatype = convolved Ca^2+^ events with 2 s kernel, behavioral datatype = head-centric data, temporal binning width = 50 ms, OA–CA data matching = nonmatched; shuffling method = randperm.

Together, our analyses, thus far, have identified two optimal parameter settings for inferring the selectivity of individual neurons for task-relevant states using calcium imaging.

### Validation of the optimal parameter settings and applicability to unseen data

Our analyses thus far have yielded the conclusion that two parameter settings, namely, #20 and #21, are optimal, in that they yield the most reliable inferences about the selectivity of individual neurons for task-relevant states using calcium imaging.

To assess how “tolerant” this conclusion is to variability within the data, we adopted a standard validation procedure using bootstrap resampling. Then to assess how “applicable” this conclusion is when “new data” are considered—often referred to as generalization error—we adopted a standard holdout cross-validation procedure. We describe these two analyses next.

**Figure 5.**
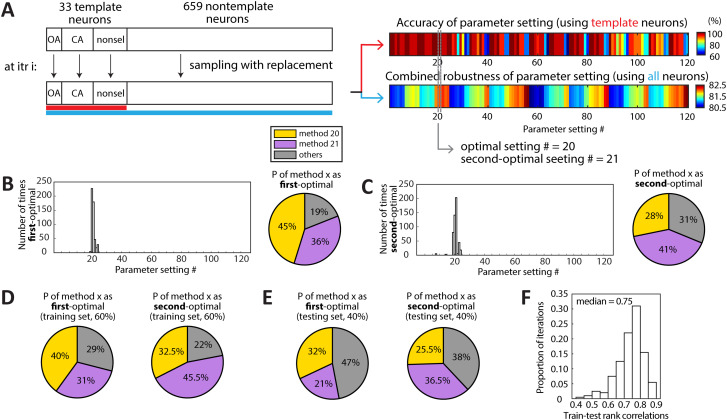
Validation of the identified optimal parameter settings. ***A–C***, Bootstrap validation. ***A***, Schematic illustration of the bootstrapping validation procedure, showing a single iteration as an example (Materials and Methods). ***B***, Left, Number of iterations identifying each parameter setting # as the first-optimal across 500 bootstrap iterations. Right, Probability of identifying either method 20 or method 21 as the first-optimal method across 500 bootstrap iterations. ***C***, Left, Number of iterations identifying each parameter setting # as the second-optimal across 500 bootstrap iterations. Right, Probability of identifying either method 20 or method 21 as the second-optimal method across 500 bootstrap iterations. ***D–F***, Holdout cross-validation. ***D***, Left, Probability of identifying either method 20 or method 21 as the first-optimal method across 200 iterations using the training set (60% of the full data; Materials and Methods). Right, Probability of identifying either method 20 or method 21 as the second-optimal method across 200 iterations using the training set. ***E***, Left, Probability of identifying either method 20 or method 21 as the first-optimal method across 200 iterations using the testing set (40% of the full data). Right, Probability of identifying either method 20 or method 21 as the second-optimal method across 200 iterations using the testing set. ***F***, Distribution of correlations between training and testing datasets (200 iterations) of the ranking of all the parameter settings. The median rank correlation was 0.75.

From the original dataset of 692 neurons, we first created one bootstrap sample (with replacement) also of size 692, such that the fractions of template neurons (of each type) and nontemplate neurons matched the original data (Fig. 5*A*, left). Using this sample, we calculated the accuracy and robustness for each of the parameter settings as before (Fig. 5*A*, right), and identified the optimal setting(s). This constituted one bootstrap iteration. We repeated this procedure for 500 iterations and analyzed the identities of the resulting 500 optimal (and second-optimal) parameter settings (Fig. 5*A*, right; gray lines).

The optimal settings from this bootstrap approach were largely identical to the top two settings identified previously (Fig. 5*B*). Specifically, either parameter setting #20 or #21 was the optimal one in the majority (81%) of all iterations (#20 in 45% and #21 in 36% of all iterations; Fig. 5*B*). Similarly, either parameter setting #20 or #21 was the second-optimal one in the 69% of all iterations (#20 in 28% and #21 in 41% of all iterations; Fig. 5*C*). Put another way, parameter setting #20 was either the optimal or second-optimal in 73% of all the iterations, and parameter setting #21 was either the optimal or second-optimal in 77% of all the iterations (in contrast, the next best parameter settings, corresponding to the blue dots in Fig. 4*E*, were either first- or second-optimal for a substantially smaller fraction of the iterations: parameter settings #19 in 17% and #23 in 12%). These results revealed that our conclusion of the optimality of parameter settings #20 and #21 was robust to variability within the data.

Next, we performed holdout cross-validation by randomly splitting our dataset into 60% training and 40% testing sets. In each split, both template neurons (of each type) and nontemplate neurons were proportionally assigned to the training and testing sets. For each iteration, we calculated the accuracy and robustness of every parameter setting and identified the optimal setting(s) as before, using the training and testing data separately. This procedure was repeated for 200 iterations.

We first examined the identities of the resulting 200 optimal (and second-optimal) parameter settings. When using the training set, either parameter setting #20 or #21 was the optimal one in the majority (71%) of all iterations (#20 in 40% and #21 in 31% of all iterations; Fig. 5*D*). Similarly, either parameter setting #20 or #21 was the second-optimal one in the 78% of all iterations (#20 in 32.5% and #21 in 45.5% of all iterations; Fig. 5*D*). When using the testing set, either #20 or #21 was again most often selected as optimal (53% of all iterations; #20 in 32% and #21 in 21% of all iterations; Fig. 5*E*) and as second-optimal in 62% of iterations (#20 in 25.5% and #21 in 36.5%; Fig. 5*E*). These results revealed that parameter settings #20 and #21 remain consistently competitive even when subsets of the data are used.

Additionally, we compared the ranking of all parameter settings (based on accuracy and robustness) between training and testing data in each iteration by calculating the rank correlation. The median rank correlation across iterations was 0.75, indicating a moderately high correspondence (Fig. 5*F*). This result revealed that the overall ordering (“goodness”) of parameter combinations is well preserved from training to testing data, with top-performing settings consistently remaining among the best, whereas poorly performing settings consistently remain poor.

Taken together, our results from bootstrapping and holdout procedures supported the legitimacy of our conclusion that parameter settings #20 and #21 are the preferred ones for reliably assessing the selectivity of individual neurons using calcium imaging.

### Generality of optimal parameter settings across methods of assessing selectivity

**Figure 6.**
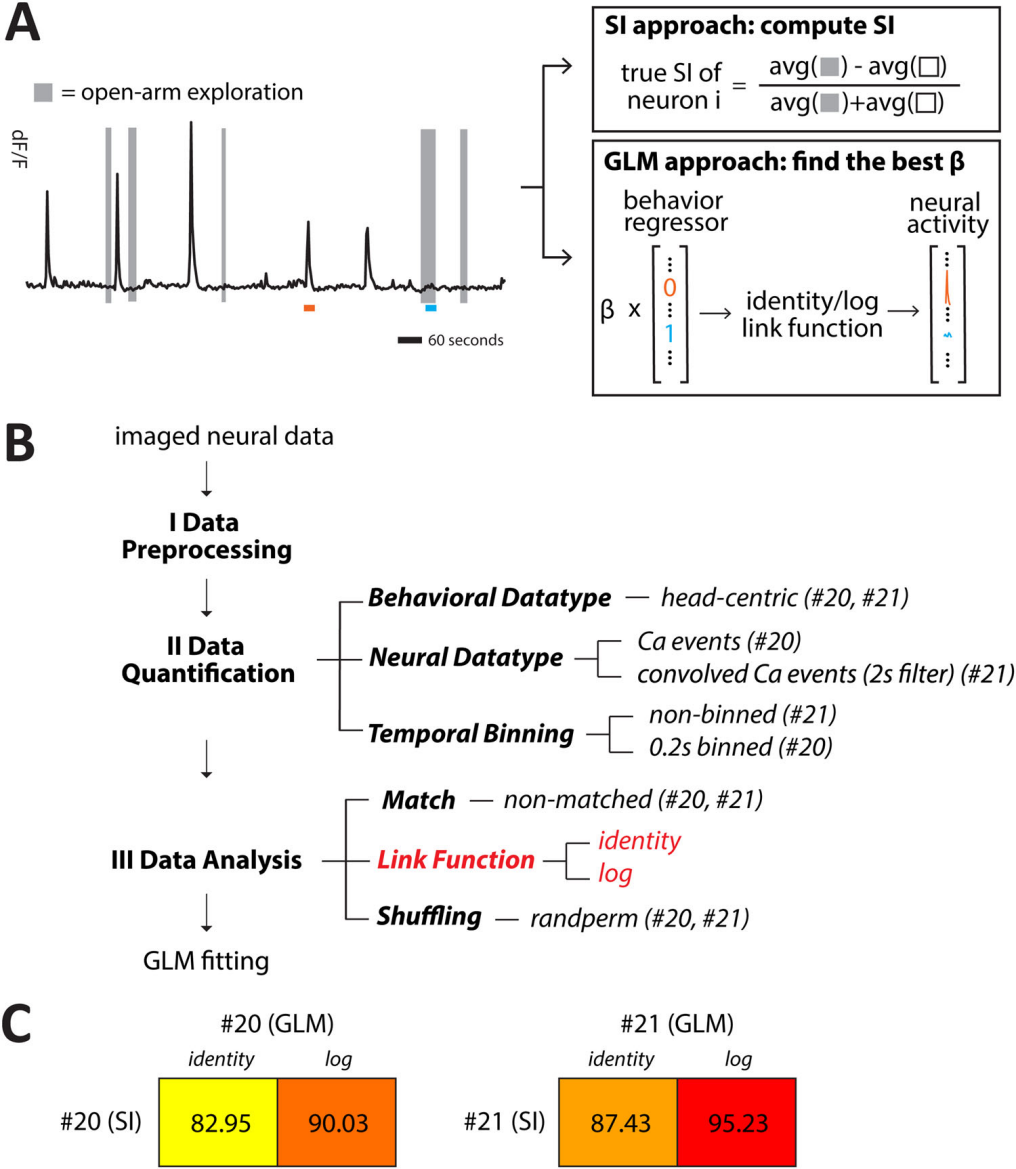
Generality of optimal parameter settings across procedures for inferring encoding preferences of neurons. ***A***, Left, Calcium transients of an example neuron. The time spent in open arms is shown by gray bars. Right, Schematic comparison of the SI approach, which uses average activity, and the GLM approach, which models instantaneous activity. Values of the calcium trace and behavioral regressor at two example time points (marked in orange and blue) illustrate the differences in input to each method. ***B***, For the regression procedure, the initial data preprocessing steps are identical to those before (Fig. 2*A*), the data quantification parameters are identical save for the temporal binning parameter having levels that are more gradual (binned at sampling step, binned at 4 × sampling step = 100 ms), and the data analysis step of fitting a regression model involves the same parameters as above (Fig. 3*A*), with the addition of a link function parameter (with values of the identity function, or a log function). The statistical approach also uses a permutation test—comparing the actual value(s) of beta against a null distribution(s) obtained by randomly permuting associations. ***C***, Selectivity label consistency, defined as the percentage of neurons assigned the same selectivity label under both the SI and GLM approaches, for the two optimal parameter settings identified via the SI approach. The two settings are additionally coupled with either an identity or a log link function when using the GLM approach.

How general are our conclusions? The selectivity index, or more broadly, the comparison of the “average” activity of a neuron to different task states over the behavioral session, represents a widely used procedure for characterizing the encoding preferences of individual neurons (Adhikari et al., 2011; Jimenez et al., 2018; Gründemann et al., 2019; Lee et al., 2019; Fig. 6*A*). However, an alternate procedure, adopted in several recent calcium imaging studies (Pinto and Dan, 2015; Chen et al., 2016; Runyan et al., 2017; Ponserre et al., 2022), involves the use of the instantaneous, “time-varying” activity of a neuron. Here, task states are treated as explanatory variables within a linear or generalized regression model (LM or GLM) to account for the activity of a neuron at each moment (Fig. 6*A*). The resulting regression coefficients (betas) are used to characterize the neuron’s encoding preference (Pinto and Dan, 2015; Runyan et al., 2017; Ponserre et al., 2022). We, therefore, wondered to what extent the optimality of parameter settings #20 and #21 holds beyond the selectivity index procedure to the regression procedure as well.

To test this, we incorporated the parameter levels from each of the settings #20 and #21 into the regression procedure and coupled these with either an identity link function (linear modeling, LM; Altman and Krzywinski, 2015) or a log link function (Poisson generalized linear modeling, GLM; Nelder and Wedderburn, 1972; McCullagh and Nelder, 1989; Fig. 6*B*; Materials and Methods). We then compared the neuronal selectivities inferred in each case against those inferred via the selectivity index procedure.

We found that the most consistent results between the selectivity index procedure and the regression procedure were obtained with parameter setting #21 coupled with the log link function (GLM; Fig. 6*C*). In contrast, parameter setting #20 did not generalize, with only weakly consistent results between the selectivity index and the regression procedures.

Based on the entirety of our results—from optimality analyses, cross-validation, and generality analysis—parameter setting #21 (2 s convolved events binned at the sampling rate, head-centric data, randperm, nonmatched) emerged as the broadly optimal choice of parameter levels. This setting yields reliable, i.e., accurate and robust, conclusions about the encoding preferences of individual neurons to task states that are also invariant to the two major procedures used to compute selectivity.

## Discussion

In this study, we investigated in detail the extent to which parameter choices along the data processing and analysis pipeline impact inferences about the encoding preferences of individual neurons to distinct task-relevant states, a question of major interest in systems and behavioral neuroscience.

Our findings address challenges related to the neural datatype faced by studies due to two essential elements: the use of calcium imaging and occurrence of animal-controlled transitions between task states. Calcium imaging has become a frequently used technique for interrogating neural activity owing to its advantages for measuring the activity of identified neurons longitudinally, as well as for targeting cell type- and projection-specific populations. This said, the use of neural calcium dynamics offers additional challenges over the use of electrophysiological responses to assess the response properties of neurons, owing to intrinsic characteristics of calcium transients (Wei et al., 2020; Robbins et al., 2021). Because of the temporal smearing of action potentials due to the slow rise and decay kinetics of the transients and potential for saturation of the reported rate of action potentials due to nonlinearities, foundational issues including what metrics to use for accurately quantifying neural activity are not immediately obvious. Reasonable choices include the area under the curve or magnitudes of the peaks of the calcium events, but whether these different choices have different implications for the results are unclear. This issue is exacerbated in experiments in which the transitions between task states occur in a spontaneous, animal-controlled manner, as opposed to in an experimenter-controlled manner (Laviolette et al., 2005; Herry et al., 2008; Grewe et al., 2017). Unlike in the latter case, in which the experimenter can lengthen interstimulus intervals to ensure that the decay of the calcium transient following an action potential during one state does not “spillover” into the next state, no such a priori steps can be taken in naturalistic behaviors. In such situations, measurements of “area under the curve” to quantify responsiveness may suffer from inaccuracies if transitions frequently occur after a calcium event has been initiated but before the calcium dynamics have fully decayed to baseline. Whereas this argument may suggest that magnitudes of discrete calcium events may be a better metric, especially in the context of animal-controlled state transitions, the choice is not obvious. This is because continuous traces are preferable over discrete events for analyses of neural ensemble activity (as smoothed data mitigate deleterious effects of sparseness also in high dimensions; Kass et al., 2003; Grewe et al., 2017; Zhang and Li, 2018; Courtin et al., 2022). In other words, there is intrinsic uncertainty about the metric that is best suited for quantifying neural activity from calcium dynamics, an issue we addressed here.

We also addressed the challenges associated with subsequent choices of (nonparametric) methods typically used to assess statistical significance of inferences about neuronal selectivity. Random permutation (*randperm*; Jimenez et al., 2018; Geva et al., 2023) and circular shifting (*circshift*; Gründemann et al., 2019; Frost et al., 2021; Fustiñana et al., 2021) are the two most commonly used methods for obtaining the shuffled dataset (see also Frost et al., 2021). *Circshift* largely preserves the local structure of the calcium signal, whereas *randperm* does not. However, the degree of randomness achieved by *circshift* shuffling is less than that achieved by *randperm* shuffling. Thus, randomizing the occurrence of events sufficiently while still retaining their local structure is a nontrivial challenge. Also nontrivial is the assessment of how choices of shuffling methods affect inferences of neuronal selectivity.

Beyond issues related to neural data and calcium imaging, we also tackled the problem of whether the head-centroid or the body-centroid serves as a better unidimensional metric for quantifying the spatial location of freely moving animals engaged in naturalistic tasks. Although both are reasonable, and body-centroid is the more commonly used metric (Jimenez et al., 2018; Courtin et al., 2022; Geva et al., 2023), certain behavioral gestures can only be unambiguously described using head-centric characterization. For instance, nose-pokes to peer over the edge of a ledge in an arena and head extensions to peer across the boundary between two distinct task-relevant zones are well-characterized using head-centric coordinates but may not be detectable using body-centric ones. How this choice may affect the assessment of neural encoding properties is not immediately obvious.

To address these challenges, here, we developed a novel, data-driven approach to systematically compare the “quality” of inferences produced by each combination of parameter values. In developing this approach, we were guided by the reasoning that identifying neurons selective for one state versus another must depend solely on the objective disparity in their neural responses, i.e., on the intrinsic characteristics of the neural signal, rather than on subjective parameter choices. Using this approach, we identified two optimal parameter combinations (#20 and #21) that produced accurate as well as reliable inferences of neuronal selectivity. This conclusion held true despite variability within the dataset and survived a cross-validation procedure, with settings #20 and #21 emerging as the preferred ones to infer the encoding preferences of individual neurons using the well-established selectivity index procedure; there was a 91% consistency between the predictions obtained using these two settings. Notably, however, when we tested these two settings in the context of a different procedure for inferring neuronal encoding preferences, namely, the recently popular time-dependent regression-based procedure, we found that only setting #21 survived the test of generality. The plausibility of this finding lies in the observation that setting #21 utilizes continuous neural data (convolved calcium events), whereas setting #20 utilizes discrete neural data (discrete calcium events). Thus, the optimal parameter combination for producing reliable inferences about neuronal selectivity across procedures is: “calcium events convolved with a 2 s exponential decay filter, head-centric animal position data, 50 ms binning of data, animal-controlled dataset sizes for task states, and randperm shuffling for statistical testing.”

This said, we must consider that our results were obtained by imaging CaMKII neurons in the mPFC using GCaMP6f. For the above-mentioned reasons relating to the kinetics of genetically encoded calcium indicators and nonlinearities in the transduction of firing rates into calcium signals, we cannot rule out completely the possibility that the parameter setting identified here may not be the optimal one for other calcium indicators, in other cell types, or in other layers/brain areas (containing neurons with different firing statistics). Nonetheless, it represents a promising starting point. Especially considering the widespread use of GCaMP6f and the imaging of CAMKII+ neurons in cortical areas, the optimal method identified here may be of more general use than at first view. Importantly, the methodology we have developed here itself possesses significant value: it prescribes a recipe for determining the optimal parameter settings under conditions different from the ones considered here. Applying this recipe to other circumstances, especially to neurons with different firing statistics or cellular nonlinearities, offers promise for the identification of the optimal parameter setting for drawing reliable inferences in that context.

In conclusion, our findings establish a hitherto missing foundation for objective and reliable characterization of neuronal encoding preferences to (animal-controlled) task states using calcium imaging.

## Data availability

Analysis code used to generate the figures is available at Zenodo at the following link: https://doi.org/10.5281/zenodo.15708398. Materials used will be made available upon reasonable request to the corresponding author.

## Synthesis

Reviewing Editor: Michael McDannald, Boston College Decisions are customarily a result of the Reviewing Editor and the peer reviewers coming together and discussing their recommendations until a consensus is reached. When revisions are invited, a fact-based synthesis statement explaining their decision and outlining what is needed to prepare a revision will be listed below. The following reviewer(s) agreed to reveal their identity: Michael McDannald. Note: If this manuscript was transferred from JNeurosci and a decision was made to accept the manuscript without peer review, a synthesis may not be available.

We solicited a third review of your manuscript, in addition to the prior reviews from JNeurosci. The reviewer was positively inclined, though brought up some higher level points. If you feel that addressing these points will improve the manuscript, you are welcome to edit the manuscript.
