# Supplementary figures and images for: Reliable Inference of the Encoding of Task States by Individual Neurons Using Calcium Imaging
Source: eNeuro. 2026 Jan 26;13(1):ENEURO.0378-25.2025. doi: 10.1523/ENEURO.0378-25.2025 (PMC12854197; doi:10.1523/ENEURO.0378-25.2025)

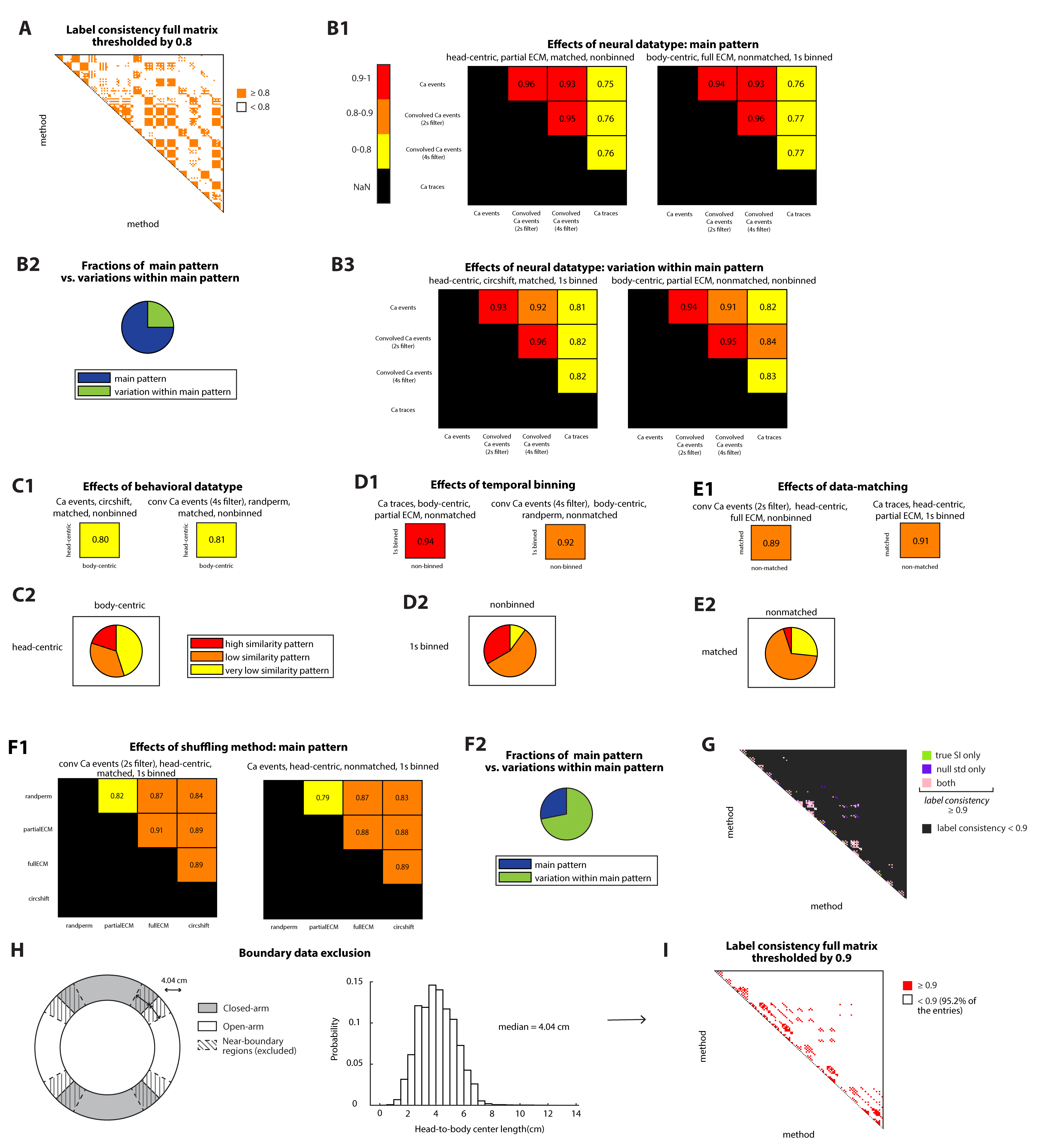

Supplement: Figure 3-1 — Effects of parameters on label consistency, and mechanisms of inconsistency. (A) Thresholded label consistency matrix (from Fig. 3A). Orange shading (consistency values significantly greater than 0.8); significance computed using bootstrap confidence intervals (CIs) for each matrix entry (Methods). (B1-F1) Submatrices of parameter effects. Example pairwise label consistency submatrices illustrating the main effects of each parameter. Red shading (as in B), orange shading (medium effect, consistency values less than 0.9 but significantly greater than 0.8), and yellow shading (large effect, consistency values not significantly greater than 0.8); see Methods. (B2-F2) Pie charts summarizing parameter main effects and interaction effects between parameters. For C2-E2, pie charts visualize the distribution of consistency levels (red, orange, yellow) across all submatrices representing the parameter’s effect. For example, in the matched-nonmatched pie chart (E2), percentages of red/orange/yellow entries were calculated from 64 submatrices (squares) related to “effects of data-matching.” The dominance of orange entries indicates that matched and nonmatched generally had a medium effect across all conditions. Interaction effects. Pie charts also highlight interaction effects between parameters. In the absence of interactions, pie charts would display homogeneous colors (e.g., all red or orange). However, most pie charts demonstrate mixed colors, indicating significant interactions among parameters. For B2 and F2, the pie charts visualize the distribution of the main pattern (in B2, very low consistency between calcium traces and other neural data types, and high consistency among other pairs; in F2, very low consistency between randperm and partial ECM, and low consistency among other pairs) as well as variations within the main pattern. The presence of these variations indicates interaction effects between parameters. (B3) Example pairwise label consistency submatrices illu [file eneuro-13-ENEURO.0378-25.2025-s003.tif]

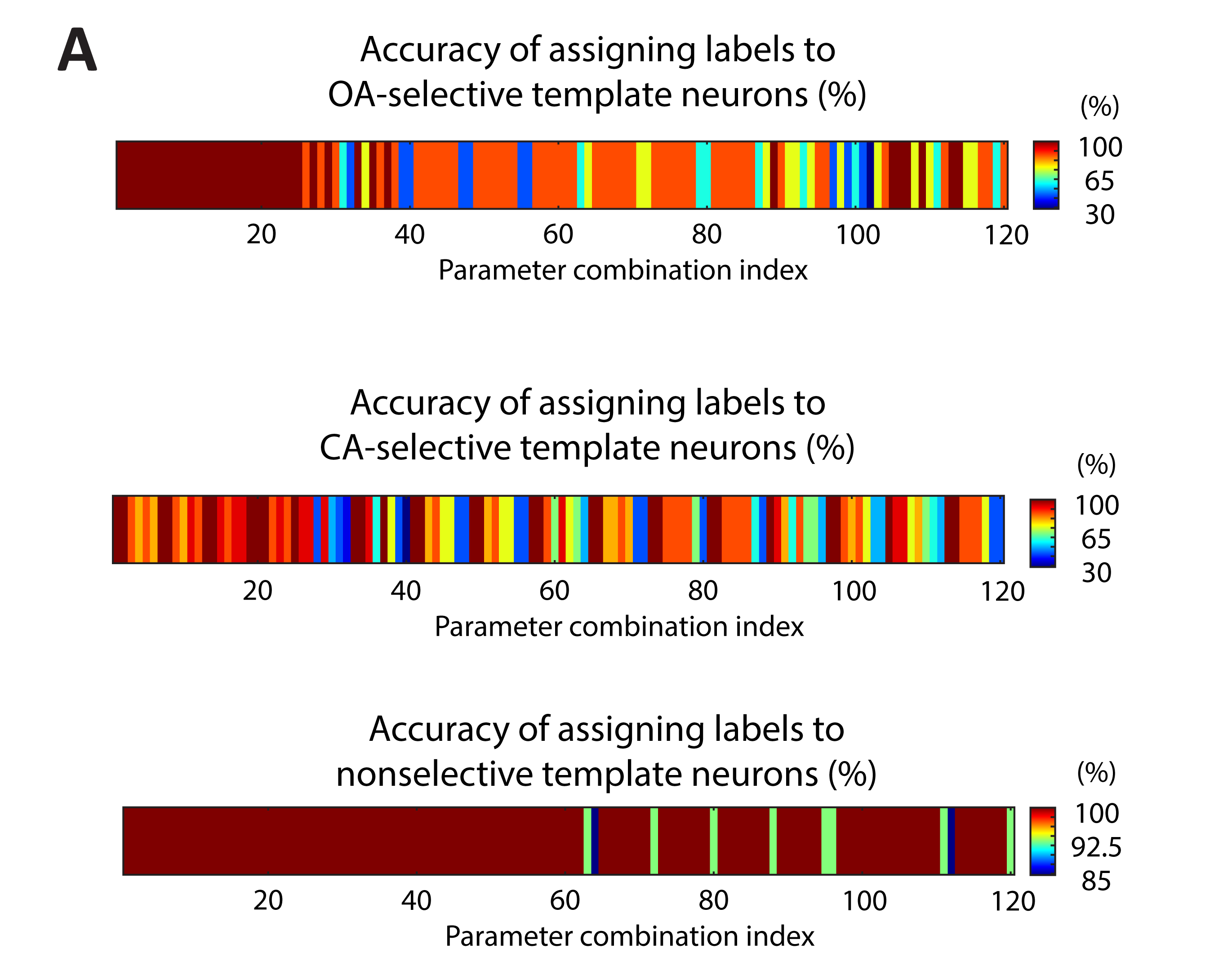

Supplement: Figure 4-1 — Accuracy and robustness analysis of parameter settings. (A) Breakdown of Fig. 4B (parameter settings accuracy) by template neuron type. For each parameter setting, the percentage of OA-selective, CA-selective, and non-selective template neurons that were correctly assigned to their respective selectivity labels is shown. Download Figure 4-1, TIF file. [file eneuro-13-ENEURO.0378-25.2025-s004.tif]
